# COVID-19-Associated Severe Aplastic Anemia in an 18-Month-Old Child: A Case Report

**DOI:** 10.7759/cureus.98419

**Published:** 2025-12-03

**Authors:** Hoor Hamidoglu, Roa'a Abu Tawileh, Hanan N Abouelkhel

**Affiliations:** 1 Department of Basic Medical Sciences, College of Medicine, University of Sharjah, Sharjah, ARE; 2 Department of Pediatrics, Alexandria University, Alexandria, EGY; 3 Department of Pediatric Hematology, Al Qassimi Women's and Children's Hospital, Sharjah, ARE

**Keywords:** bone marrow failure, hematopoietic stem cell transplantation, pancytopenia, post-viral complications, romiplostim, severe aplastic anemia

## Abstract

The COVID-19 pandemic has significantly impacted global health since its emergence in late 2019. Established evidence indicates an increasing incidence of hematologic abnormalities caused by COVID-19, such as aplastic anemia. Only a few pediatric cases have been reported. We present a previously healthy 18-month-old boy with fever, cough, diarrhea, rash, and ear discharge for one week. He was febrile, tachycardic, drowsy, with bilateral purulent ear discharge, petechiae, and cervical lymphadenopathy. Blood tests showed severe pancytopenia with high inflammatory markers, and SARS-CoV-2 PCR was positive. Bone marrow biopsy revealed severe aplastic anemia (<10% cellularity), and genetic testing excluded inherited causes. He was treated with blood transfusions and romiplostim. After one year of supportive management, the patient showed a partial response and improving counts, so bone marrow transplantation was deferred, with continued monitoring for sustained hematologic recovery or relapse.

## Introduction

Since late 2019, SARS-CoV-2 has had a major global impact, with illness ranging from mild upper respiratory symptoms to multisystem disease. In children, most infections are mild; fever and cough predominate, though severe presentations - including pneumonia/acute respiratory distress syndrome (ARDS), septic shock, acute cardiac failure, and multisystem inflammatory syndrome in children (MIS-C) - occur in a minority [1,2]. Hematological abnormalities, notably lymphopenia, thrombocytopenia, and elevated D-dimer, are increasingly reported, and rare cases of bone marrow failure, including aplastic anemia (AA), have raised concern [2].

Aplastic anemia is a life-threatening bone marrow failure syndrome that targets hematopoietic stem cells, diagnosed by the presence of peripheral pancytopenia and hypocellular bone marrow. It can be categorized into inherited and acquired forms. A significant proportion of acquired cases are idiopathic, while others are post-viral infections, post-drugs, and toxins [3,4]. In pediatrics, viral infections have been implicated as the most common cause of acquired aplastic anemia [3,4].

AA is further classified into three categories based on the severity of the drop in blood counts to determine prognosis and choose a treatment plan. A severe AA is defined as bone marrow cellularity <25% with at least two of the following: absolute neutrophil count of <0.5 × 10⁹/L, platelet count of <20 × 10⁹/L, or absolute reticulocyte count of <60 × 10⁹/L. Very severe AA includes severe AA criteria, but with an absolute neutrophil count <0.2 × 10⁹/L. Moderate AA (non-severe AA) is diagnosed when peripheral blood counts do not fulfill the criteria of severe and very severe AA [5]. Although the role of SARS-CoV-2 remains unclear, there have been few reports of acquired aplastic anemia in children triggered by SARS-CoV-2 [6-9].

## Case presentation

A previously healthy 18-month-old boy presented to the emergency department with a one-week history of fever, mild cough, nasal congestion, diarrhea, rash, and right-sided ear discharge. Despite three days of oral Augmentin prescribed for otitis media, he didn’t improve. He refused oral feeding during fever episodes, but his weight didn't change. The child is fully immunized according to the United Arab Emirates (UAE) immunization schedule. On admission, the child was febrile (38.9°C), tachycardic (134 bpm), mildly tachypneic, and drowsy. He had bilateral purulent ear discharge, enlarged tonsils, small bilateral anterior cervical lymphadenopathy, and petechial rash. The chest was clear to auscultation and percussion, with good air entry bilaterally and no added sounds. The abdomen was soft, non-tender, not distended, and had no organomegaly.

Capillary blood gases (CBG) analysis revealed a pH of 7.36, PCO_2_: 41.5 mmHg, PO_2_: 20.8 mmHg, HCO_3_-: 23 mmol/L, base excess: -2.2, glucose: 5.6 mmol/L, lactate: 1.25 mmol/L, and a hemoglobin of 5.2 g/dL. Laboratory findings showed pancytopenia: hemoglobin 6.9 g/dL, platelets 4,000/µL, absolute neutrophil count 0.18 x 10³/µL, and absolute reticulocyte count 24.30 x 10³/µL, fulfilling the definition of severe aplastic anemia [5]. He had elevated inflammatory markers - C-reactive protein 258 mg/L and procalcitonin 1.24 ng/mL. Lactate dehydrogenase (LDH), liver enzymes, alkaline phosphatase (ALP), and coagulation profile were all normal. The direct antiglobulin test was negative.

Laboratory testing ruled out (influenza A and B, hepatitis A, B, and C, cytomegalovirus {CMV}, Epstein-Barr virus {EBV}, parvovirus B19, dengue virus, and HIV). However, SARS-CoV-2 PCR was positive. Ear pus, blood, and urine cultures were clear. Chest X-ray showed bilateral accentuated bronchovascular markings, and CT head showed right temporal scalp swelling with impression of mastoiditis. Blood film revealed pancytopenia, predominantly normocytic normochromic RBCs, and leukocytopenia with absolute neutropenia and several reactive lymphocytes. Bone marrow biopsy confirmed severe aplastic anemia with suppressed trilineage hematopoiesis and less than 10% cellularity. Hemophagocytic lymphohistiocytosis (HLH) and Langerhans cell histiocytosis (LCH) were ruled out (insufficient criteria for HLH diagnosis; S100 stain for LCH was negative). Infiltration with malignancy was ruled out. Genetic testing (whole exome sequencing) ruled out inherited bone marrow failure syndrome and primary immunodeficiency.

In the ER, the patient was managed with an IV normal saline bolus at 20 mL/kg, IV paracetamol at 15 mg/kg, and IV ceftriaxone at 75 mg/kg (septic dose). Upon admission, he received a platelet transfusion (10 mL/kg), followed by a packed red blood cells (PRBCs) transfusion (15 mL/kg). During his one-month stay, the child required seven platelets and two PRBC transfusions, a short trial of thrombopoietin mimetic therapy (romiplostim), and IV ceftriaxone at 75 mg/kg (septic dose).

A COVID-19 PCR test was done before his discharge day and came out negative. The child was eventually discharged with a prescription for fluconazole and trimethoprim prophylaxis, advised to avoid crowded places and contact with sick people. Parents were counseled regarding the risk of bleeding and advised to avoid NSAID suppositories and IM injections.

**Figure 1:**
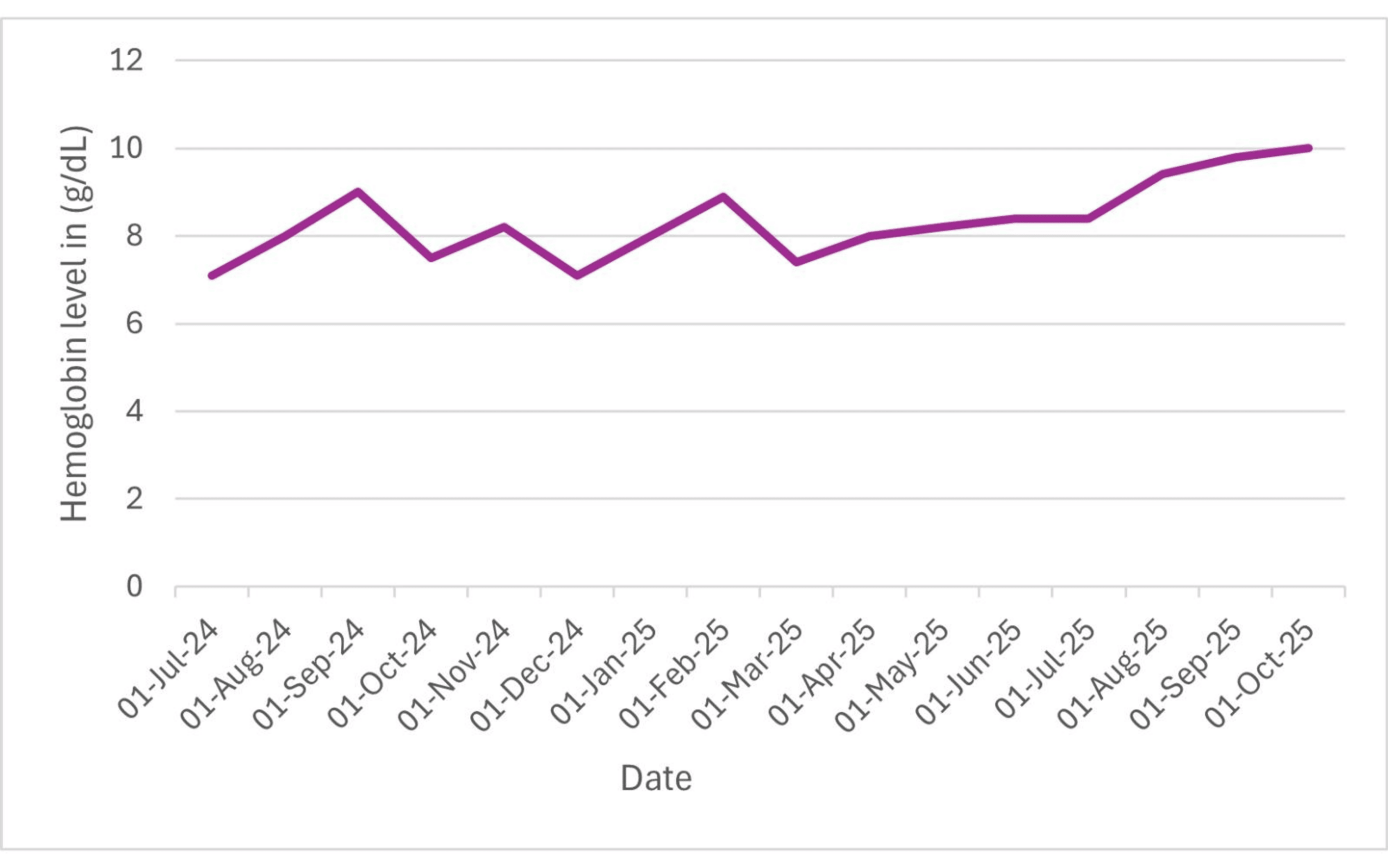
Hemoglobin levels fluctuated during early treatment, but over time they gradually increased following immunosuppressive therapy with ATG, cyclosporine, and prednisolone, indicating progressive hematologic stabilization. ATG: antithymocyte globulin

**Figure 2:**
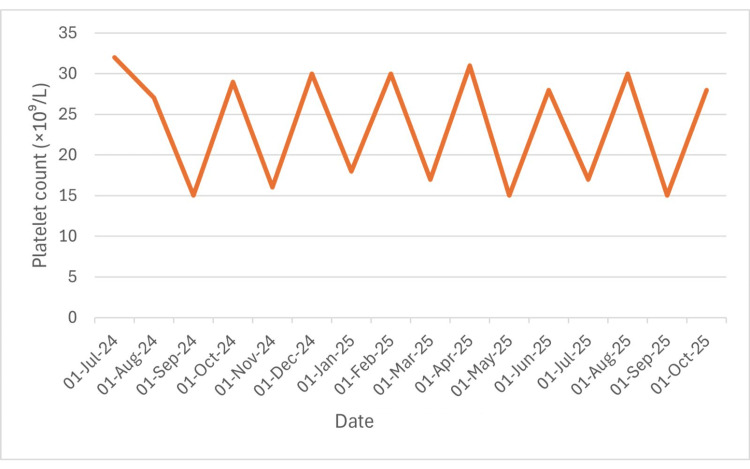
Trend of platelet counts showed repeated fluctuations with transient rises following thrombopoietin and transfusions, and subsequent declines, consistent with ongoing transfusion dependence despite partial hematologic recovery.

**Figure 3:**
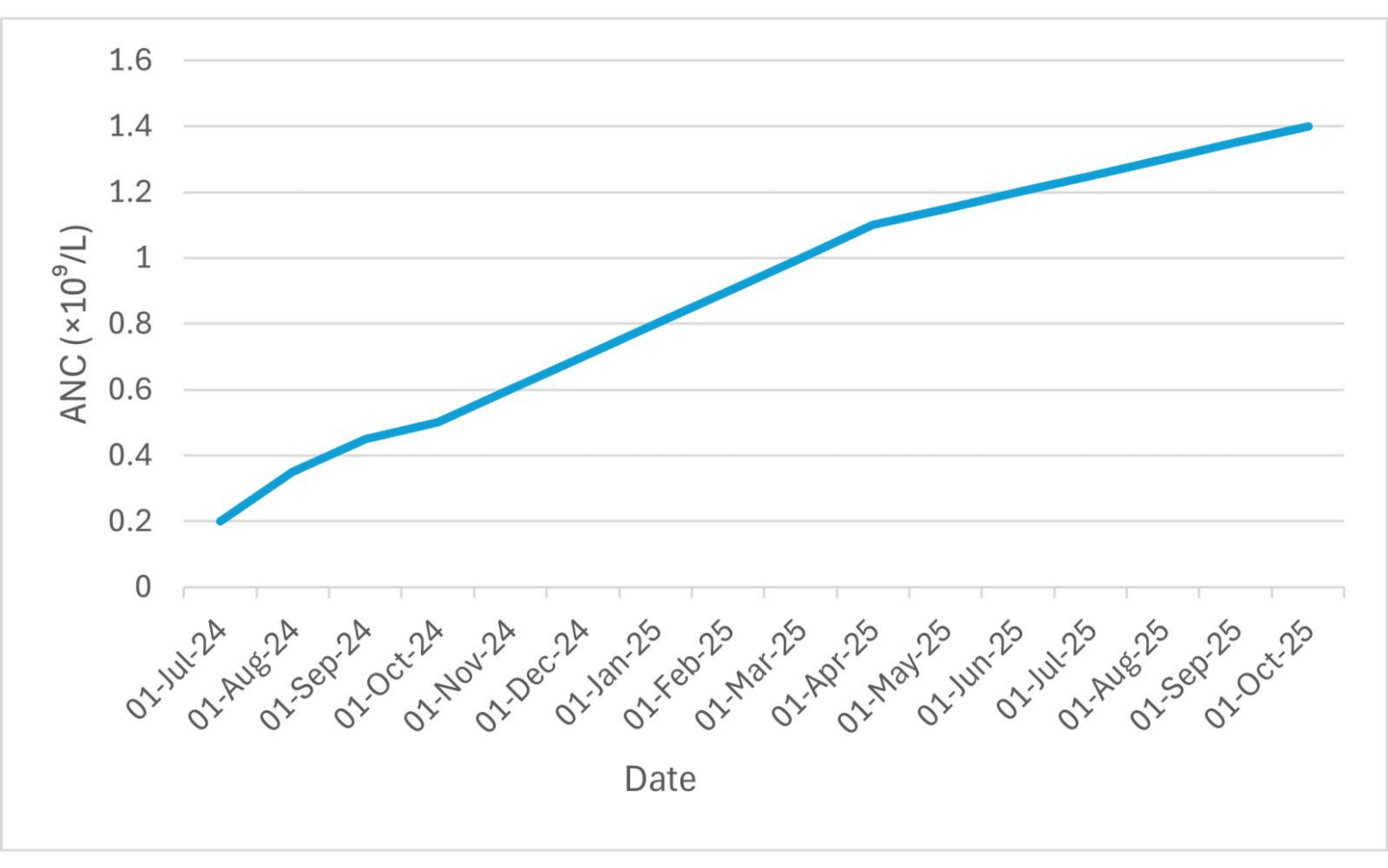
Progressive improvement in ANC from July 2024 to October 2025, indicating hematologic recovery following supportive therapy and resolution of prior neutropenia. ANC: absolute neutrophil count

After one year of supportive therapy with PRBCs and platelet transfusions, the patient received a course of horse antithymocyte globulin (ATG) combined with cyclosporine and a short tapering course of prednisolone. The patient’s hemoglobin and absolute neutrophil count (ANC) stabilized by September-October 2025, indicating a partial hematologic response. However, he continues to require weekly platelet transfusions (Figures 1-3). Given the patient’s partial response and improving counts, bone marrow transplantation was deferred, with continued monitoring for sustained hematologic recovery or relapse.

## Discussion

The pathophysiology of aplastic anemia involves a complex interplay of factors - immune-mediated mechanisms, genetic predispositions, and environmental factors - leading to destruction or dysfunction of hematopoietic stem cells (HSCs) in the bone marrow [3,4]. The most widely accepted theory is that aplastic anemia is primarily immune-mediated bone marrow failure in which environmental insults (e.g., viral infection, drugs) precipitate an oligoclonal expansion of cytotoxic CD8⁺ T cells that target HSPCs. Cytokines released by cytotoxic T cells, including IFN-γ, TNF-α, and TGF-β, play a crucial role in the inhibition and apoptosis of HSCs, which leads to bone marrow aplasia [3,4].

SARS-CoV-2 has been reported to cause pancytopenia, but the underlying mechanism remains unclear. One proposed explanation is a direct viral effect on the bone marrow, as HSPCs express ACE2 receptors that allow viral entry; experimental studies show that SARS-CoV-2 can infect erythroid precursors, which may lead to pure red-cell aplasia in both children and adults [6,10]. Another possible mechanism is immune-mediated injury, in which SARS-CoV-2 triggers the activation of cytotoxic CD8⁺ T cells and a proinflammatory cytokine response. High levels of IFN-γ and TNF-α have been shown to suppress marrow activity by inducing apoptosis of hematopoietic stem and progenitor cells (HSPCs) and impairing maturation. Together, these mechanisms may contribute to COVID-19-related bone marrow failure [6-8,10].

Upon comparing our case with the existing literature, we found no notable differences in the clinical presentation of documented cases. Our patient presented with fever, respiratory and gastrointestinal symptoms, and a petechial rash. Other studies have also reported a broad spectrum of symptoms, including headaches, sore throat, bleeding, purpura, severe fatigue, chest tightness, and bone pain [1,2]. While the temporality of disease onset following COVID-19 remains unclear, several reports support a delayed-onset pattern of hematological decline following infection. In one pediatric series, AA developed 15 days to several weeks after infection clearance [7]. Another report describes a child with persistent pancytopenia two weeks post-recovery, progressing to marrow hypocellularity [8]. Our case demonstrates a similar progression, with a notable decline in total blood count persisting after recovery from COVID-19.

Reported management strategies vary, reflecting the lack of pediatric-specific guidelines. Our approach involved platelet and red-cell transfusions and a short course of romiplostim. Other studies describe antivirals, immunomodulators, and combinations of transfusions with steroids, alongside referral for HSCT, with practice guided by severity and donor availability [5,8].

Outcomes in pediatric COVID-19-associated AA are variable; some improve with supportive care or thrombopoietin-receptor agonists, while others develop severe complications [7,8]. Our patient started improving after one year of continuous transfusions; as a result, bone marrow transplantation was deferred. In the present case, we couldn't identify the exact mechanism of COVID-19-induced aplastic anemia due to the lack of viral PCR analysis of the bone marrow and the unavailability of a cytokine study.

## Conclusions

To date, documented cases of COVID-19-induced pancytopenia in pediatric patients remain limited. This case adds to the growing body of evidence that SARS-CoV-2 can exert significant hematological effects, even in very young children. It highlights the potential association between SARS-CoV-2 and the development of severe acquired aplastic anemia in pediatric patients. While transient pancytopenia has been recognized as a possible complication of COVID-19, the occurrence of persistent acquired aplastic anemia warrants careful monitoring and prompt referral for specialized management. Further studies are needed to establish causality, discover long-term outcomes, and develop evidence-based management strategies.
